# Retraction: One pot green preparation of Seabuckthorn silver nanoparticles (SBT@AgNPs) featuring high stability and longevity, antibacterial, antioxidant potential: a nano disinfectant future perspective

**DOI:** 10.1039/d2ra90062a

**Published:** 2022-06-17

**Authors:** Thiyagarajan Kalaiyarasan

**Affiliations:** Defence Institute of High Altitude Research (DIHAR), DRDO Leh-Ladakh-194101 J&K India advances-rsc@rsc.org

## Abstract

Retraction of ‘One pot green preparation of Seabuckthorn silver nanoparticles (SBT@AgNPs) featuring high stability and longevity, antibacterial, antioxidant potential: a nano disinfectant future perspective’ by Thiyagarajan Kalaiyarasan *et al.*, *RSC Adv.*, 2017, **7**, 51130–51141, https://doi.org/10.1039/C7RA10262C.

I, the undersigned author, hereby wholly retract this *RSC Advances* article due to the following instances of matched/similar images that have been identified that weaken this article, which occurred due to honest human errors.

Following the previous publication of a correction to correct errors in Fig. 1, 3, 7 and 9, further instances of duplicating images have been identified within the corrected Fig. 3 and 7, as well as additional errors in the original article, that undermine this article.

In Fig. 3 of the correction notice:

The panel for freshly prepared *S. enterica* MTCC-3219 2 μg ml^−1^ 10^−10^ is identical to the panel for *S. typhirmurium* MTCC-3224 4 μg ml^−1^ 10^−5^ after one year of storage, to the panel 1 h treated with SBT@AgNPs in Fig. 7, and to the panels *E. coli* MTCC No. 62 6 μg ml^−1^ and 8 μg ml^−1^ in Fig. 5 of ref. 1.

The panel for freshly prepared *S. typhirmurium* MTCC-3224 6 μg ml^−1^ 10^−10^ is identical to the panel for *S. typhirmurium* MTCC-3224 6 μg ml^−1^ 10^−10^ after one year of storage and to the panel 0.5 h treated with SBT@AgNPs in Fig. 7.

The panel for freshly prepared *S. typhirmurium* MTCC-3224 4 μg ml^−1^ 10^−5^ is identical to the panel for freshly prepared *S. enterica* MTCC-3219 6 μg ml^−1^ 10^−10^.

The panel for freshly prepared *S. typhirmurium* MTCC-3224 2 μg ml^−1^ 10^−10^ is identical to the panel for *S. typhirmurium* MTCC-3224 6 μg ml^−1^ 10^−5^ after one year of storage.

The panel for freshly prepared *S. typhirmurium* MTCC-3224 4 μg ml^−1^ 10^−10^ is identical to the panel for *S. typhirmurium* MTCC-3224 2 μg ml^−1^ 10^−10^ after one year of storage.

The panel for *S. enterica* MTCC-3219 4 μg ml^−1^ 10^−5^ after one year of storage is identical to the panel for *S. enterica* MTCC-3219 6 μg ml^−1^ 10^−10^ after one year of storage.

In Fig. 3a, a′, c and c′ of the original article, the error bars are erroneous.

In Fig. 4 of the original article, both the panels for *S. typhirmurium* are identical.

Thiyagarajan Kalaiyarasan and Vijay K. Bharti responded to all enquiries and submitted data related to the above concerns. However, to avoid any future ambiguity to the readers, the article is retracted.

Vijay K. Bharti and O. P. Chaurasia were informed about the retraction of the article but have not responded.

Signed: Kalaiyarasan Thiyagarajan

Date: 1/6/2022

Retraction endorsed by Laura Fisher, Executive Editor, *RSC Advances*

## Supplementary Material
